# Microsurgical robotic system enables the performance of microvascular anastomoses: a randomized in vivo preclinical trial

**DOI:** 10.1038/s41598-023-41143-z

**Published:** 2023-08-27

**Authors:** Gerardo Malzone, Giulio Menichini, Marco Innocenti, Alberto Ballestín

**Affiliations:** 1 Division of Plastic, Reconstructive and Microsurgery, CTO Careggi University Hospital, Florence, Tuscany, Italy; 2 IV Clinica Ortoplastica, IRCCS Istituto Ortopedico Rizzoli, Bologna, Italy; 3 Tumor Microenvironment Laboratory, Institut Curie, Orsay - Paris, France; 4 Microsurgery Department, Jesús Usón Minimally Invasive Surgery Center, Cáceres, Spain

**Keywords:** Reconstruction, Translational research, Surgical oncology

## Abstract

Technical advances in microsurgery have enabled complex oncological reconstructions by performing free tissue transfers, nerve and lymphatic reconstructions. However, the manual abilities required to perform microsurgery can be affected by human fatigue and physiological tremor resulting in tissue damage and compromised outcomes. Robotic assistance has the potential to overcome issues of manual microsurgery by improving clinical value and anastomoses’ outcomes. The Symani Surgical System, a robotic platform designed for microsurgery, was used in this *in-vivo* preclinical study using a rat animal model. The tests included anastomoses on veins and arteries performed by microsurgeons manually and robotically, with the latter approach using Symani. The anastomoses were assessed for patency, histopathology, and execution time. Patency results confirmed that the robotic and manual techniques for venous and arterial anastomoses were equivalent after anastomosis, however, the time to perform the anastomosis was longer with the use of the robot (p < 0.0001). Histological analysis showed less total average host reaction score at the anastomotic site in robotic anastomosis for both veins and arteries. This study demonstrates the equivalence of vessel patency after microsurgical anastomoses with the robotic system and with manual technique. Furthermore, robotic anastomosis has proven to be slightly superior to manual anastomosis in terms of decreased tissue damage, as shown by histological analysis.

## Introduction

Microsurgery allows high-precision dissection and suturing of very small anatomical structures using microscopic magnification and dedicated instrumentation. Technical advances in microscopes, manual instrumentation, and sutures allow microsurgeons to perform complex trauma and oncological reconstructions by performing free tissue transfers, nerve reconstructions, and lymphatic microsurgery^1^. Using these microsurgical techniques, different surgical specialties today can perform operations that would otherwise be impossible^2^. Dexterity and technical accuracy are paramount for the successful practice of microsurgery. Therefore, extensive training and clinical experience are essential to overcome a steep learning curve^3–5^. Moreover, the manual abilities required to perform microsurgery can be affected by human fatigue, and undesired involuntary movements such as physiological tremor may result in tissue damage and compromise outcomes^6^. Robotic assistance has the potential to overcome such issues by filtering physiological tremor^7^.

Robotic surgery expanded rapidly over the past two decades and is widely used in several surgical specialties^8^. Robotic systems were introduced initially in endoscopy and laparoscopy to improve surgeon capabilities and procedure reproducibility^9^. The better recoveries and shorter hospital stays of patients increased the number of laparoscopic and robotic-assisted procedures and reduced open procedures avoiding invasive laparotomies. Although most commercial robotic systems have limitations in the areas of motion scaling and precision, some attempts at using them in microsurgery have been made.

The da Vinci Surgical System (Intuitive Surgical, Inc., Sunnyvale, CA, USA) designed for laparoscopic minimally invasive surgery has been tested in some microsurgical procedures^10^. These attempts demonstrated the benefit of wristed instruments and revealed their limitation in size and tips, which were designed for laparoscopy. Moreover, this system provides limited visual magnification and poor resolution associated with low scaling factors^11^. The MUSA system (Microsure, Eindhoven, The Netherlands) has been designed to overcome the limitations of the human hand and the gaps left by the da Vinci. MUSA uses manual instrumentation mounted on a suspension ring attached to the operating table, aiding in stabilizing movements of the microsurgeon by filtering tremor and scaling down motions^12^. The system has been tested in preclinical studies and demonstrated equivalency with manual microsurgical technique in clinical cases of lymphatic anastomoses^13^.

Recently, a new robotic device (Symani Surgical System, MMI, Pisa, Italy) designed for microsurgical procedures, received CE certification in 2019. The system features dedicated wristed microinstruments, overcoming the limitations of large diameter instruments such as those used with the da Vinci system, and high-motion precision^14–16^. The system has recently been evaluated in bench tests demonstrating the feasibility of performing precise microsutures and anastomoses in synthetic vessels^14^.

The purpose of this multicenter preclinical study was to evaluate the quality of microsurgical anastomoses performed with the new robotic system by comparing vessel patency, histological outcomes, and execution times, with the anastomoses performed created with the traditional manual technique.

## Results

### Patency evaluation

**Figure 1:**
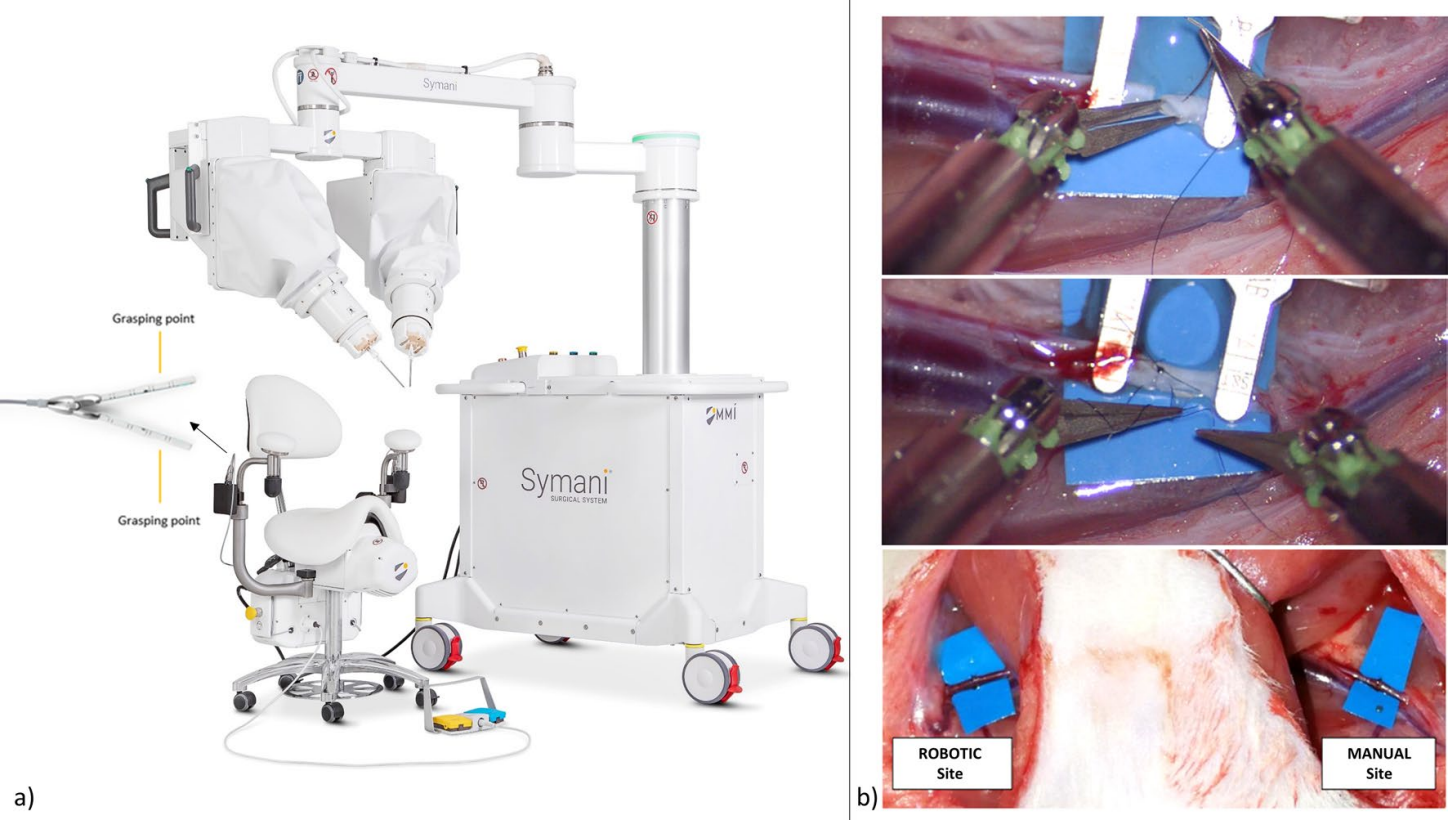
(**a**) Overview of the Symani Surgical System and close-up view of the manipulator. (**b**) Surgical scenario during a robotic microsurgery procedure and end product after manual and robotic anastomoses in the rat femoral arteries.

Microsurgical anastomoses were performed on rat femoral vessels with the manual technique and with the new robotic system (Fig. 1). Patency of operated veins after clamp removal (T0) averaged 91% for the robot and 100% with the manual technique (p = 0.15). After one week (T1W), the robotic patency rate remained constant at 91%, while manual patency decreased to 77% (p = 0.22). The average patency rate for arteries at T0 was 94% for robotic procedures and 100% for manual technique (p = 0.32); at T2W, patency decreased to 91% for the robotic group and to 85% for the manual group (p = 0.55).

### Histological analysis

Histological analysis of the vein samples showed that all examined anastomosis sites performed robotically and manually presented one or more of the following histological findings with different degrees of severity: endothelial loss, intima proliferation, presence of fibrin clots attached to the endothelium, intraluminal thrombosis, inflammatory reaction of the tunica intima, media and/or adventitia. This inflammatory reaction frequently appeared together with neovascularization and fibrosis. The host tissue reaction at the suture sites mostly consisted of a combination of inflammatory cell types, with presence of giant cells mostly associated with sutures.

**Figure 2:**
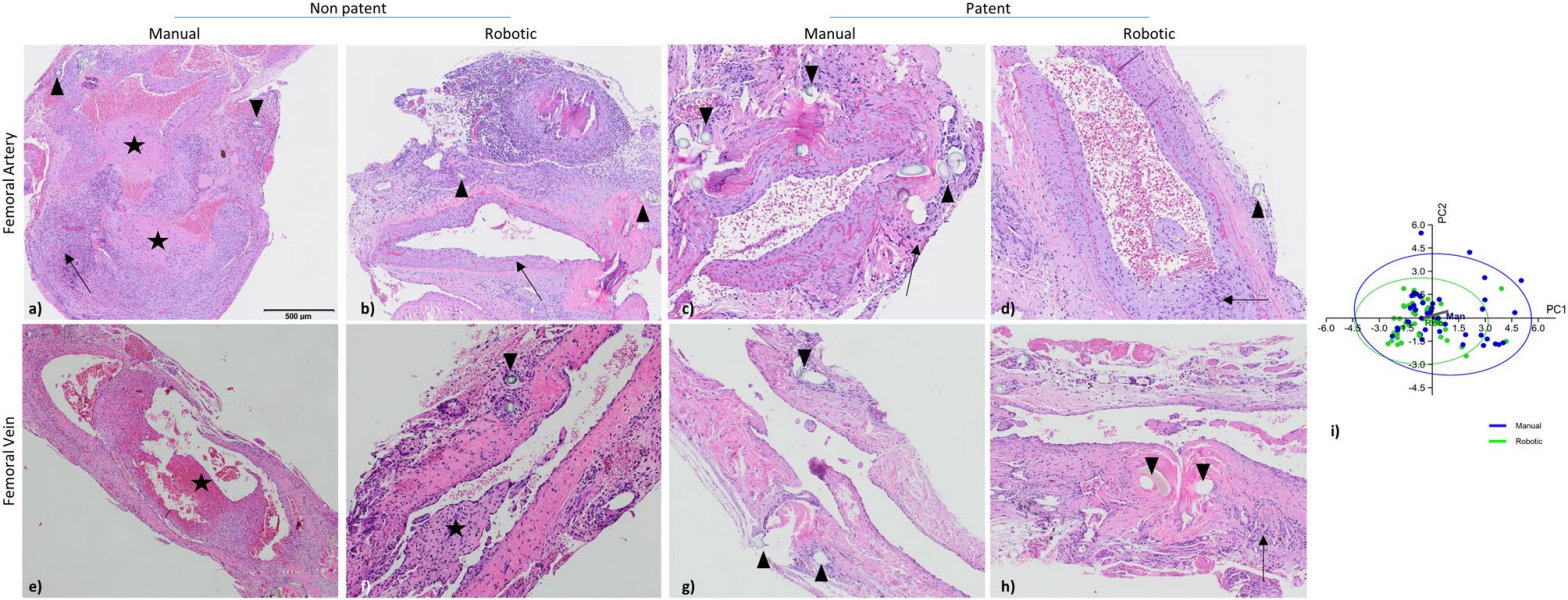
Representative histological samples of non-patent and patent vessels after microsurgical anastomosis on femoral arteries and veins. The asterisks indicate the thrombosis, the triangles the sutures and the arrows the inflammatory infiltrate. (**a**) Representative image of a non-patent manual arterial anastomosis: Presence of high inflammatory infiltrate and thrombosis. (**b**) Robotic arterial anastomosis with presence of inflammatory infiltrate. (**c**) Patent manual anastomosis with inflammatory infiltrate. (**d**) Patent robotic with really low inflammatory infiltrate comparing previous images. (**e**) Non-patent venous anastomosis, with the presence of a chronic thrombus markedly occluding and expanding the vein lumen. (**f**) Robotic venous anastomosis with presence of a chronic thrombus partially occluding the vein lumen. (**g**) Patent manual vein anastomosis with low inflammatory infiltrate. (**h**) Patent robotic anastomosis with low inflammatory infiltrate. (**i**) Principal component analysis (PCA) results for Robotic (Rob, green dots) versus Manual (Man, blue dots) procedures. The grey line connects centroids (Rob and Man) of the two groups and highlights the degree of separation.

In the arterial samples, one or more of the following histological findings were detected with different degrees of severity: endothelial loss, proliferation of the tunica intima associated with smooth muscle proliferation or proteoglycans/collagen, multifocal dystrophic calcification of the arterial wall, presence of fibrin clots attached to the endothelium and/or intraluminal thrombus varying in size. These fibrin clots and thrombi partially or completely blocked the artery varying from acute to chronic chronicity. The inflammatory reaction of the vascular wall was detected either at the tunica intima or within the adventitia, while the host tissue reaction at the suture sites mostly consisted of a mix of inflammatory cells, with the presence of giant cells mostly associated with the suture stitches. Also, fibroblastic proliferation and fibrosis were commonly observed at the anastomosis sites with neovascularization. Figure 2 shows examples of histological samples of two arteries (Fig. 2a,b) and two veins (Fig. 2c,d) sutured with either manual or robotic technique, while Table 1 summarizes the total host reaction scores in vein and artery studies. All parameters analyzed were lower in robotic procedures compared to manual for both arteries and veins. Indeed, the total average score was 36.5 and 31.6 for vein anastomoses performed manually or with the robot respectively, while it was 37.5 and 26.3 for arteries regarding manual and robotic approaches respectively.

**Table 1:** Histological results of veins and arteries samples for manual and robotic procedures.

Summarized histopathology evaluation	Veins		Arteries	
	Manual	Robotic	Manual	Robotic
Host reaction sum per group	546	471	834	606
Average vessel reaction	10.25	6.8	12.1	6.4
Average vessel inflammation	7.1	6.4	6.2	3.8
Average inflammatory/host reaction at the suture site	19.2	18.4	19.6	16.1
Total average score (vessel reaction + vessel inflammation + tissue reaction at the suture site)	36.5	31.6	37.9	26.3

PCA results (Fig. 2e) demonstrated that manual and robotic anastomoses were separated by three main histological variables that were all indicators of tissue trauma: endothelial loss, fibrin/platelet thrombus, and intimal proliferation. The first two PCs highlighted the degree of separation between the two groups, also indicated by the grey line linking the corresponding centroids. Robotic surgery had lower variation in histopathological outcomes and a greater reproducibility (Fig. 2e). Manual surgeries had a greater variability and were characterized by higher values for histological variables due to tissue trauma.

### Execution time

**Figure 3:**
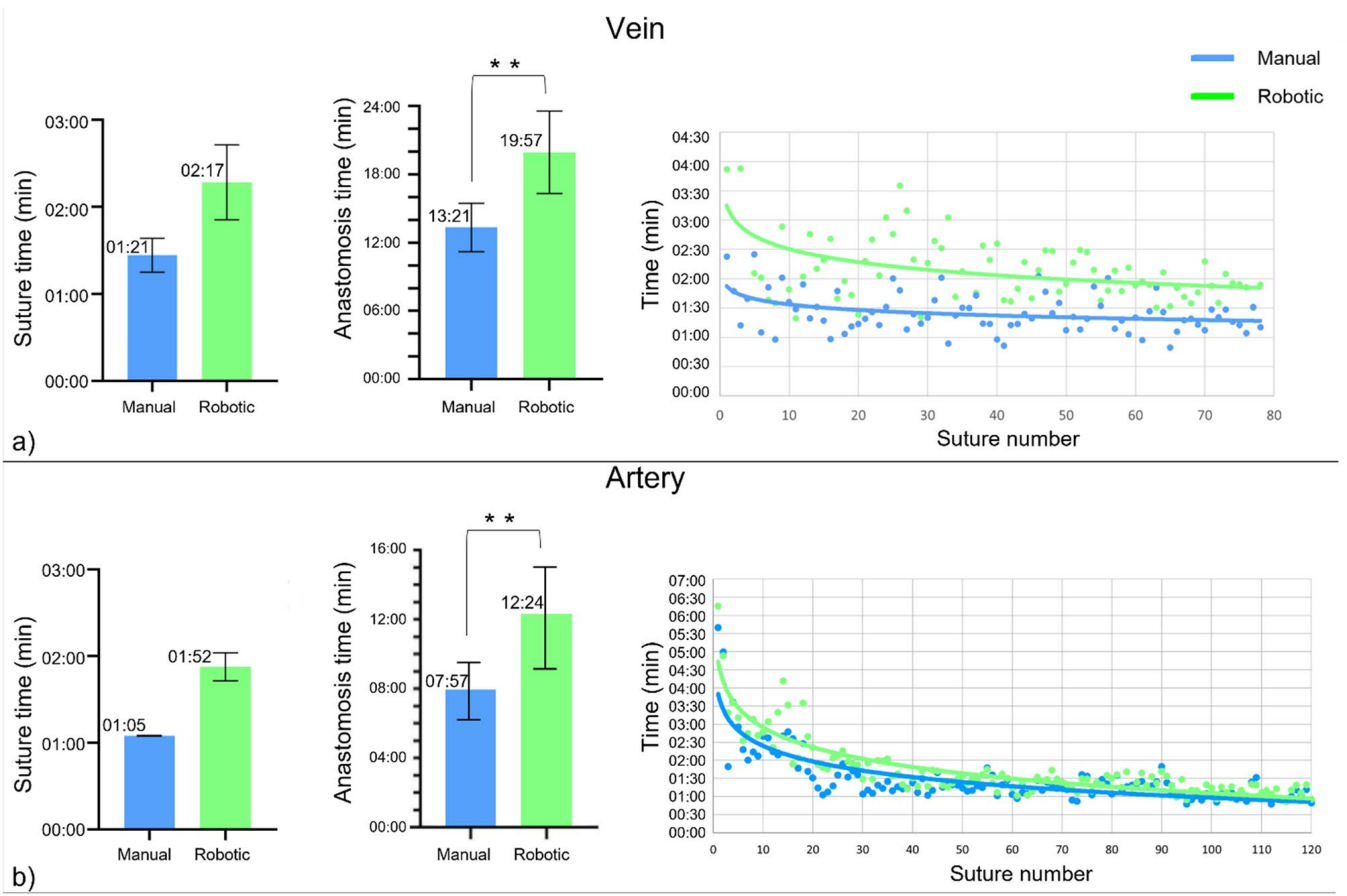
Average suture time, average anastomosis time, and learning curves for (**a**) vein and (**b**) artery studies.

Figure 3 reports the average suture time, the average anastomosis time, and learning curves for both manual and robotic anastomoses in the vein (Fig. 3a) and artery (Fig. 3b) studies. Execution time for suturing was longer with the robotic system than with the manual technique. Robotic mean times were 136.9 ± 25.78 s for veins (02:17 min) and 112.7 ± 9.7 s for arteries (01:52 min), which compares to mean times of 81.3 ± 11.4 s for veins (1:21 min), and 65.0 ± 0.2 s for arteries (01:05 min). Thus, vein anastomosis time was 19:57 min for the robotic group versus 13:21 min for the manual vein group (p < 0.0001). In case of arteries, the anastomosis time was 12.24 min for the robotic group, compared to 07:57 min for the manual group (p < 0.0001).

Figure 3 (right) also depicts the learning curves calculated for the suture time progression over the study for all surgeons, by plotting all consecutive sutures made during all anastomoses. A trend of decreasing suture time as the study progressed was visible in the robotic curve, after the performance of several robotic anastomoses, the times of consecutive sutures became more similar, indicating a progressive familiarization with the robotic system. In contrast, the manual suturing times were steady.

There were no significant differences related to vascular size in vessels used in this study among groups. The mean rat femoral vein diameter was 1.03 ± 0.25 mm for the robotic procedures and 1.09 ± 0.31 mm for the manual ones. Moreover, the mean number of stitches was 10 for both the manual and the robotic procedures. Similarly, the rat femoral artery diameters were similar between robotic and manual groups (0.71 ± 0.15 mm and 0.70 ± 0.10 mm respectively). The mean number of stitches was 8 in both robotic and manual arterial anastomosis.

There was one unexpected death; the animal died two hours after the intervention after a normal awakening from anesthesia. This animal showed patent anastomoses in both robotic and manual sites at T0.

## Discussion

This in vivo preclinical study presents for the first time data that demonstrate the feasibility and accuracy of using the Symani Surgical System in arterial and venous microvascular anastomoses based on patency, time for anastomosis execution, and histology of operated vessels.

In this study, there were no blood vessel diameter differences in the vein and artery studies nor in robotic and manual procedures. This demonstrates that patency, histological and execution time results were not influenced by vessel-size differences, which we consider essential to delineate the conclusions from our preclinical study.

Patency results and Chi-squared tests confirmed that the robotic and manual techniques for venous and arterial anastomoses were equivalent in terms of patency after anastomosis, as had already been observed with the use of Musa, the other microsurgery robot on the market^11^. At the chronic time points (T1W and T2W in vein and artery respectively), the robotic patency rate was higher in robotic cases than with the manual technique, probably due to a less delicate vessel handling and therefore the presence of thrombosis in some of the vessels operated manually, however differences were not statistically significant. Use of the robot was associated with a longer time to execute an anastomosis than manual. A robotic venous anastomosis took 6 min longer, on average, than manual execution, while a robotic arterial anastomosis took on average 4 min longer. However, the speed of execution was not the primary endpoint in these studies nor an indicator of anastomosis quality. Independent of vessel type, learning curves demonstrated that after about 70 sutures the users reached a plateau of suture execution time with the robot. The rapid improvement of execution times was observed after performing several robotic anastomoses, as already described. As such, the technical aspects of robotic microsurgery may be rapidly obtained by surgeons even those without previous experience in microsurgery or robotic surgery^8^. The relatively low learning curve may be an important driver of microsurgical robotic adoption, as surgeons may become adept with robotic microsuturing more quickly compared to using the conventional manual microsurgery technique^17^.

Regarding execution times, our results were similar to those using the Musa robot, since the time to perform the anastomosis was longer with the robot^11^, however, we observed the improvement on the quality of the microsurgical performance according to the histology results.

Histological analysis showed less total average host reaction score at the anastomotic site in robotic anastomoses. The total average score was lower in robotic procedures for both vein and artery studies. This may be due to the ability of robotic systems to improve surgery by extending human capabilities by reducing surgeons' movements and scaling micro-movements, and eliminating physical tremor^13,14,18^. The conditions of the tissue indicated that robotic anastomoses had less inflammation, less intimal hyperplasia and lower total average host reaction compared to tissue from manual microsuturing. These phenomena are hallmarks of vessel trauma, thus, the trauma seen in manual anastomoses may lead to an increased thrombosis rate. Nevertheless, these improved robotic microsurgical results need to be verified in clinical studies.

There were no clinical signs of thrombosis or ischemia during the survival period. Even in animals in which the femoral vessel anastomosis were not patent, hindlimb blood flow was not totally compromised since three collateral routes through intramuscular networks in the quadriceps femoris muscle, biceps femoris muscle and the medial thigh muscles including the medial hamstring muscles and adductor muscles remained intact as previously described in literature^19,20^. Therefore, neither limp nor signs of pain were observed. With respect to animal welfare, the veterinarian supervisor carried out a daily assessment, evaluating the weight, behavior and signs of stress or pain (such as eyes closed, kyphosis, ruffled/scruffy skin coat, ears rotated out or towards back, nasal secretions). No human final points were reached for any mice and animal welfare was maintained throughout the study.

## Conclusion

The use of the Symani microsurgery robot has demonstrated equivalence with manual technique in terms of vessel patency of microvascular venous and arterial anastomoses. Furthermore, robotic anastomoses have proven to be slightly superior than manual in terms of decreased tissue damage shown by histological analysis. The safety of the robotic device is supported by a very low rate of adverse events and the device efficacy is supported by patency rates and surgeons’ ability to perform microvascular anastomosis successfully.

## Materials and methods

This *in-vivo* study was performed in a rat model, where safety and performance when executing microvascular anastomoses with Symani was assessed. Anastomosis patency rates, histological outcomes and execution times were evaluated in this study.

### The robotic system

The Symani Surgical System (Fig. 1a) is a robotic device specifically designed for microsurgical open procedures. The flexible platform uses the principles of teleoperation to provide surgeons with high precision in manipulation of very small anatomy such as vessels, nerves, and ducts. The system consists of a master console controlling the end-effectors including robotic arms on which articulated microinstruments are mounted. The robotic arms may be positioned in any anatomical region.

The Master Console is an ergonomic chair equipped with surgeon-controlled manipulators along with a footswitch. The surgeon can directly move the manipulators in the same manner as she or he would with manual instrumentation scaling down this motion to the arms and then to the robotic microinstruments. On the robotic arms of the system, articulated microinstruments (7 degrees of freedom) are installed and moved above any anatomical region. The system features motion scaling ranging between 7 × to 20 × and includes tremor filtration to increase surgical precision.

### Users

Three microsurgeons performed the arterial and venous anastomoses, they had previous microsurgery experience from 8 to 12 years. These surgeons contributed extensively to bench testing of Symani and received the training program prior to executing the anastomoses in this study. Hence, they are considered fully proficient in using the system safely and effectively.

### Animal model

This study was performed in rat femoral vessels, which are a common target in microvascular anastomosis training and research model^21,22^. The same protocol was used in the two centers where the study was carried out: the Laboratory Animal Housing Center (CeSAL), University of Florence (Florence, Italy), and the Jesús Usón Minimally Invasive Surgery Center (CCMIJU, Cáceres, Spain). Microsurgical anastomoses on femoral veins were performed on 22 Wistar rats (*Rattus Norvegicus*) with a minimum weight of 350 g. Among them, 9 rats were in a preclinical study approved in 2018 by the Italian Ministry of Health and carried out at CeSAL; while 13 rats were in a preclinical study approved in 2019 by the Ethical Committee of Animal Experimentation of the CCMIJU, where the study was performed. Microsurgical anastomoses on femoral arteries were performed on 34 Sprague Dawley rats (*Rattus Norvegicus*) with a minimum weight of 350 g. This study was approved in 2019 by the Italian Ministry of Health and carried out at the CeSAL. All methods were carried out in accordance with the specific guidelines and regulations in force in Spain and Italy, as well as in compliance with European legislation on animal welfare and protection of animals used for scientific purposes. The authors complied with the ARRIVE guidelines.

### Study design for vein and artery anastomoses

The Symani robot was placed close to the surgical table with microinstruments directed toward the rat femoral vessels (Fig. 1b). In microsurgery it is common in clinical practice to work with a microscope face-to-face: on one side of the microscope the lead surgeon (who in this study was the one who manipulated the robot during the robotic procedure) and on the opposite side of the microscope the assistant surgeon that supports during the procedure. Both the surgeon and the assistant used the microscope. The anastomoses were performed on femoral veins and arteries, the execution time was evaluated, and patency was assessed immediately after the procedure and also after one week. Histopathological characteristics of vessels were evaluated after animal sacrifice. Both the vein and artery studies were two-armed controlled trials to allow direct and unbiased comparison of the Symani-aided microanastomosis with the manual technique. Both the left and right femoral vessels of the animal were sutured. A manual (M) anastomosis was performed on one vessel, and on the other side, a robotic (R) anastomosis was performed. The treatment (M or R) was alternated with respect to the side of the animal: left or right vessel and temporal execution (M-R: first M then R; or R-M: first R then M).

Before surgery, the rats were prepared by a veterinarian with intraperitoneal anesthesia using Xylazine (5–10 mg/kg) and Zoletil100 (20–40 mg/kg) at CeSAL and with inhalatory anesthesia using Sevoflurane at CCMIJU. Rats were carefully placed in a supine position over an animal warming pad and prepared for surgery. A trichotomy of the groin areas was performed, followed by disinfection with povidone-iodine and chlorhexidine and administration of prophylactic broad-spectrum antibiotic to prevent potential post-operative infections. After that, the surgeon proceeded with skin incision and vessel preparation by using standard microsurgical instruments. Once the femoral vein or artery was exposed and isolated, a colored background was placed behind the vessel for contrast. The diameter of the vessel was measured with a digital caliber and secured with a double microvascular clamp. Then, the vessels were cut in the middle, and end-to-end microvascular anastomoses were completed using 10-0 nylon sutures (S&T AG, Neuhausen, Switzerland). After the execution of the two anastomoses (left/right rat groin, one robotic, the other manual), the skin incisions were closed with 3-0 prolene sutures (Ethicon, Pennsylvania, United States). After skin closure, the animals were monitored for physiological recovery from anesthesia and housed individually with daily animal welfare assessment.

### Patency evaluation

Patency is a primary endpoint of vein and artery studies as the restoration of vascular flow is directly related to the quality and precision of the anastomosis. Indeed, traumatic tissue handling or lack of precision in suture placement may cause a thrombotic cascade through leak or hemodynamic turbulence. The Acland’s “milking test” was used to assess anastomotic patency^23,24^. Patency outcome was binary (patent or not patent) based on surgeon’s judgment after the Acland’s milking test. Patency evaluation was performed immediately after anastomosis (T0) for both veins and arteries, with re-evaluation after one week for veins (T1W) and after two weeks for (T2W) for arteries, as complete re-endothelialization of intraluminal suture is faster in the vein than the artery^18^. The exploration of the anastomotic site was performed with the same method of analgesia, anesthesia, and incision as that at the time of intervention.

### Histological analysis

Histological analysis of the anastomosis site was conducted on vessel tissue harvested at the last patency evaluation (T1W or T2W for veins and arteries, respectively) just prior to sacrifice the animal. The tissue was collected in a closed-circuit disposable container pre-filled with 4% formaldehyde solution. The histological evaluation was performed by AnaPath Services GmbH (Liestal, Switzerland). The sections were stained with hematoxylin and eosin, as well as with Masson’s trichrome. A semi-quantitative histopathological analysis was performed for each vein and artery segment on the stained sections according to an adapted ISO 10993-6:2016(E) scoring system (Supplementary Table 1), including the following parameters: vessel reaction, vessel inflammation, inflammatory/host reaction at suture site, total average score (vessel reaction + vessel inflammation + tissue reaction at the suture site). In the analysis, a score difference of 0.0–2.9 indicates no or minimal host reaction, 3.0–8.9 indicates a slight host reaction, and 9.0–15.0 indicates a moderate host reaction while a score ≥ 15.1 indicates a severe host reaction compared to a reference material.

A Principal Component Analysis (PCA) was carried out using histological data with the aim to detect if there were different histological profiles in between all robotic and manual anastomosis carried out in all veins and arteries. PCA allows analyzing data sets that contain a variety of characteristics per observation; this allows data to be interpreted as a set while conserving joint information. PCA is used to reduce the dimensionality of the data set and allow simplified visualization of this multidimensional data. This analysis takes all the values of the histological variables as input and combines them to evaluate the degree of separation among data points in a low-dimensionality scatter plot. The three main histological variables were endothelial loss, fibrin/platelet thrombus, and intimal proliferation. We have used the first principal two components (PC1 and PC2) to plot the data in two dimensions and visually identify the closely related groups of data points: those related to robotic intervention and those related to manual intervention.

### Execution time

The time to perform single sutures, as well as the time to complete anastomoses, was recorded for vein and artery procedures as well as robotic and manual techniques. Mean values were calculated for each user. Manual and robotic anastomoses also were assessed separately.

### Statistical analysis

Statistical analyses were performed using GraphPad Prism software version 8.0 (GraphPad Software, San Diego, CA, USA). All data are expressed as the mean ± standard deviations. A Chi-squared test on patency outcomes (two-sided, confidence interval 95%) was performed to compare manual and robotic procedures. Time data are expressed as the mean ± standard deviations, and differences between means were analyzed by Student’s t-test; p-values < 0.05 were considered to indicate statistically significant differences between groups. Logarithmic regression of suture time was used for the graphical representation but further analysis was not performed. PCA was used to analyze differences between robotic and manual procedures by using confidence ellipses and their distance to emphasize groups and their separation on the scatter plots. PCA was carried out using the free software PAST (PAleontological STatistics) version 3.

### Supplementary Information

Supplementary Table 1.

## Data Availability

Correspondence and requests for materials and data should be addressed to A.B.

## References

[1] Ballestín A, Shurey S, Nikkhah D, Rawlins J, Pafitanis G (2023). Microsurgery essentials: Preconditions, instrumentation, and setup. Core Techniques in Flap Reconstructive Microsurgery: A Stepwise Guide.

[2] Tamai S (2009). History of microsurgery. Plast. Reconstr. Surg..

[3] Ghanem A (2020). International microsurgery simulation society (IMSS) consensus statement on the minimum standards for a basic microsurgery course, requirements for a microsurgical anastomosis global rating scale and minimum thresholds for training. Injury.

[4] Ramachandran S, Alrasheed T, Ballestín A, Akelina Y, Ghanem A, Selber JC (2021). Robotic microsurgical training. Robotics in Plastic and Reconstructive Surgery.

[5] Ballestín A, Akelina Y, Nikkhah D, Rawlins J, Pafitanis G (2023). Basic and advanced microvascular anastomotic techniques. Core Techniques in Flap Reconstructive Microsurgery: A Stepwise Guide.

[6] Howarth AL (2019). Work-related musculoskeletal discomfort and injury in microsurgeons. J. Reconstr. Microsurg..

[7] Veluvolu KC, Ang WT (2010). Estimation and filtering of physiological tremor for real-time compensation in surgical robotics applications. Int. J. Med. Robot..

[8] Selber JC, Alrasheed T (2014). Robotic microsurgical training and evaluation. Semin. Plast. Surg..

[9] Buess GF, Schurr MO, Fischer SC (2000). Robotics and allied technologies in endoscopic surgery. Arch. Surg..

[10] Selber JC (2010). Transoral robotic reconstruction of oropharyngeal defects: A case series. Plast. Reconstr. Surg..

[11] van Mulken TJM (2018). Preclinical experience using a new robotic system created for microsurgery. Plast. Reconstr. Surg..

[12] van Mulken TJM (2018). Robotic (super) microsurgery: Feasibility of a new master-slave platform in an in vivo animal model and future directions. J. Surg. Oncol..

[13] van Mulken TJM (2020). First-in-human robotic supermicrosurgery using a dedicated microsurgical robot for treating breast cancer-related lymphedema: A randomized pilot trial. Nat. Commun..

[14] Ballestin A, Malzone G, Menichini G, Lucattelli E, Innocenti M (2022). New robotic system with wristed microinstruments allows precise reconstructive microsurgery: Preclinical study. Ann. Surg. Oncol..

[15] Teichmann H, Innocenti M, Selber JC (2021). Development of a new robotic platform for microsurgery. Robotics in Plastic and Reconstructive Surgery.

[16] Lindenblatt N (2022). Early experience using a new robotic microsurgical system for lymphatic surgery. Plast. Reconstr. Surg. Glob. Open.

[17] Alrasheed T, Liu J, Hanasono MM, Butler CE, Selber JC (2014). Robotic microsurgery: Validating an assessment tool and plotting the learning curve. Plast. Reconstr. Surg..

[18] Boehm F (2022). Performance of microvascular anastomosis with a new robotic visualization system: proof of concept. J. Robot. Surg..

[19] Ballestin A (2018). Ischemia-reperfusion injury in a rat microvascular skin free flap model: A histological, genetic, and blood flow study. PLoS One.

[20] Kochi T (2013). Characterization of the arterial anatomy of the murine hindlimb: Functional role in the design and understanding of ischemia models. PLoS One.

[21] Pruthi N, Sarma P, Pandey P (2018). Training in micro-vascular anastomosis using rat femoral vessels: Comparison of immediate and delayed patency rates. Turk. Neurosurg..

[22] Shaughness G, Blackburn C, Ballestin A, Akelina Y, Ascherman JA (2017). Predicting thrombosis formation in 1-mm-diameter arterial anastomoses with transit-time ultrasound technology. Plast. Reconstr. Surg..

[23] Acland RD (1989). Practice Manual for Microvascular Surgery.

[24] Adams WP (2000). Patency of different arterial and venous end-to-side microanastomosis techniques in a rat model. Plast. Reconstr. Surg..

