# Author Correction: Microsurgical robotic system enables the performance of microvascular anastomoses: a randomized in vivo preclinical trial

**DOI:** 10.1038/s41598-024-59538-x

**Published:** 2024-04-18

**Authors:** Gerardo Malzone, Giulio Menichini, Marco Innocenti, Alberto Ballestín

**Affiliations:** 1 Division of Plastic, Reconstructive and Microsurgery, CTO Careggi University Hospital, Florence, Tuscany, Italy; 2 IV Clinica Ortoplastica, IRCCS Istituto Ortopedico Rizzoli, Bologna, Italy; 3 Tumor Microenvironment Laboratory, Institut Curie, Orsay - Paris, France; 4 Microsurgery Department, Jesús Usón Minimally Invasive Surgery Center, Cáceres, Spain

Correction to: *Scientific Reports* 10.1038/s41598-023-41143-z, published online 27 August 2023

The original version of this Article contained an error in Affiliation 2, which was incorrectly given as ‘IV Clinica Ortoplastica, IRCCS Istituto Ortopedico Rizzoli, Università Di Bologna, Bologna, Italy.’

The correct affiliation is listed below:

IV Clinica Ortoplastica, IRCCS Istituto Ortopedico Rizzoli, Bologna, Italy.

