# Supplementary material for: Microsurgical robotic system enables the performance of microvascular anastomoses: a randomized in vivo preclinical trial
Source: Sci Rep. 2023 Aug 27;13:14003. doi: 10.1038/s41598-023-41143-z (PMC10460789; doi:10.1038/s41598-023-41143-z)
Supplement: Supplementary file 1 — Supplementary Table 1. [file 41598_2023_41143_MOESM1_ESM.docx]

**Supplementary Table 1.** Histopathology scoring system for the semi-quantitative evaluation according to an adapted ISO 10993-6:2016(E) scoring system.

|  | Score | | | | |
| --- | --- | --- | --- | --- | --- |
|  | 0 | 1 | 2 | 3 | 4 |
| Endothelial loss (%) | 0 | Rare,1-5/hpf | 5-10/hpf | 10-20/hpf | >20/hpf |
| Surface (Fibrin/platelet thrombus) | 0 | Very small focus, covering approximately 5 endothelia | covering approximately 10 endothelia | covering approximately 10-20 endothelia | covering approximately more than 20 endothelia |
| Intima proliferation | 0 | Small focus | 2 small foci or stretched deposit covering less than 20% of lumen, height maximally twice of intima | stretched deposit covering less than 40% of lumen, height maximally three times of intima | stretched deposit covering more than 40% of lumen, height more than three times of intima |
| Smooth muscle in intima proliferation | 0 | Small focus | 2 small foci or stretched deposit covering less than 20% of lumen, height maximally twice of intima | stretched deposit covering less than 40% of lumen, height maximally three times of intima | stretched deposit covering more than 40% of lumen, height more than three times of intima |
| Proteoglycan/collagen | 0 | Small focus | 2 small foci or stretched deposit covering less than 20% of lumen, height maximally twice of intima | stretched deposit covering less than 40% of lumen, height maximally three times of intima | stretched deposit covering more than 40% of lumen, height more than three times of intima |
| Dystrophic calcification | 0 | Minimal | Slight | Moderate | Marked |
| **Calculation** | **Subtotal (x2)** | | | | |
| **Inflammation** | Score | | | | |
|  | 0 | 1 | 2 | 3 | 4 |
| Intima/media | 0 | Minimal focus | 2-3 distant foci | coalescing foci covering approx. 20% of luminal surface | inflammation affecting more the 20% of target area |
|  |  |  |  |  |  |
| Adventitia | 0 | Minimal focus | 2-3 distant foci | coalescing foci covering approx. 20% of adventitial area | inflammation affecting more the 20% of target area |
| **Calculation** | **Subtotal (x2)** | | | | |
| **Inflammatory / Host reaction at suture site** | Score | | | | |
|  | 0 | 1 | 2 | 3 | 4 |
| Polymorphonuclear cells | 0 | Rare,1-5/hpf | 5-10/hpf | Heavy infiltrate | Packed |
| Lymphocytes | 0 | Rare,1-5/hpf | 5-10/hpf | Heavy infiltrate | Packed |
| Plasma cells | 0 | Rare,1-5/hpf | 5-10/hpf | Heavy infiltrate | Packed |
| Macrophages | 0 | Rare,1-5/hpf | 5-10/hpf | Heavy infiltrate | Packed |
| Giant cells | 0 | Rare,1-2/hpf | 3-5/hpf | Heavy infiltrate | Sheets |
| Necrosis | 0 | Minimal | Mild | Moderate | Severe |
| **Calculation** | **Subtotal (x2)** | | | | |
| Fibrosis | 0 | Narrow band | Moderately thick band | Thick band | Extensive band |
| Neovascularization | 0 | Minimal capillary proliferation, focal, 1 to 3 buds | Groups of 4 to 7 capillaries with supporting fibroblastic structures | Broad band of capillaries with supporting fibroblastic structures | Extensive band of capillaries with supporting fibroblastic |
| **Calculation** | **Subtotal** | | | | |
| **Calculation** | **Total sample count** | | | | |
|  | **Host reaction Group Total** | | | | |
|  | **Host reaction Average** | | | | |
